# Use of the Otago Exercise Programme for Fall Prevention in Adults After Stroke: A Best‐Evidence Summary for Clinical Nursing Practice

**DOI:** 10.1111/jnu.70157

**Published:** 2026-11-11

**Authors:** Huang Yuqing, Li Yuan, Ye Hongmei, Huang Canrong, Song Yufan, Gong Qin

**Affiliations:** ^1^ School of Nursing Fujian University of Traditional Chinese Medicine Fuzhou China; ^2^ Affiliated Rehabilitation Hospital of Fujian University of Traditional Chinese Medicine Fuzhou China; ^3^ Fujian Provincial Key Laboratory of Rehabilitation Technology Fuzhou China

**Keywords:** accidental fall, evidence summary, evidence‐based nursing, Otago Exercise Programme, stroke

## Abstract

**Introduction:**

Falls are common after stroke, but evidence on the Otago Exercise Programme (OEP) specifically for post‐stroke fall prevention remains limited. This evidence summary aimed to identify, appraise, and synthesize the best available evidence on the OEP for fall prevention in adults after stroke.

**Design:**

A systematic, implementation‐oriented evidence summary guided by the 6S evidence model and the Joanna Briggs Institute evidence appraisal methods.

**Methods:**

Two reviewers independently completed study selection, methodological quality appraisal, and evidence extraction. A systematic search of guideline repositories, clinical decision resources, and bibliographic databases was conducted, covering the period from database inception to May 2026. Eligible sources included direct OEP‐related studies and relevant stroke rehabilitation or fall‐prevention guidance used to inform clinical implementation.

**Results:**

In total, 14 publications were included, and 18 best‐evidence statements were generated across 6 domains: fall risk assessment, exercise intervention principles, core OEP components, exercise prescription and implementation, safety management and health education, and intervention effects. Evidence suggests that the OEP may improve balance function, lower‐limb muscle strength, mobility, fall‐related self‐efficacy, activities of daily living, and quality of life in adults after stroke. However, evidence regarding actual fall incidence and fall‐related injuries remains insufficient.

**Conclusion:**

The OEP can be integrated as an exercise‐based component of comprehensive fall‐prevention care for adults after stroke. Implementation should be guided by fall risk assessment, pre‐exercise safety screening, individualized exercise prescription, patient and caregiver education, and continuity of care. The findings of this evidence summary provide practical guidance for nurses and rehabilitation professionals implementing OEP‐based fall prevention across hospital, community, and home settings.

**Clinical Relevance:**

This evidence summary provides nurses and rehabilitation professionals with a practical, safety‐oriented framework for implementing the OEP across hospital, community, and home settings to address post‐stroke fall risk.

**Protocol Registration:**

PROSPERO (CRD420261418397).

## 1 Introduction

Stroke is one of the leading causes of death and long‐term disability in China. According to the Global Burden of Disease Study 2021, stroke continues to account for a substantial proportion of global mortality and morbidity, and its overall burden has increased over the past three decades (GBD 2021 Stroke Risk Factor Collaborators. 2024). Given the large population of patients with stroke in China, the demand for post‐stroke rehabilitation and long‐term management has grown substantially (Zhu et al. 2025). During post‐stroke rehabilitation, the quality of functional recovery directly influences patients' quality of life and the potential for community reintegration. However, several complications may impede the rehabilitation process. Falls are among the most common and clinically significant adverse events. Post‐stroke falls may result in fractures, traumatic brain injury, rehospitalisation, and activity restriction. These events may also exacerbate the fear of falling, reduce adherence to rehabilitation training, and impair quality of life (Chen et al. 2023; Zhang et al. 2025).

The Otago Exercise Programme (OEP) is a home‐based, progressive, multimodal exercise intervention. It primarily comprises lower‐limb strengthening exercises, balance training, and walking exercises. Evidence suggests that the OEP may improve fall‐related self‐efficacy, activities of daily living, and quality of life in older patients with stroke (Park and Chang 2016). The American Heart Association/American Stroke Association Guidelines for Adult Stroke Rehabilitation and Recovery also emphasize functional training and the restoration of balance and mobility after stroke (Winstein et al. 2016). Nevertheless, direct evidence on the OEP for fall prevention in patients with stroke remains limited. Existing studies are constrained by small sample sizes, inconsistent intervention protocols, and insufficient reporting of fall incidence outcomes.

Although previous reviews and evidence summaries have examined exercise‐based fall prevention or OEP‐related interventions, evidence specifically organized for OEP‐based fall prevention in adults after stroke remains limited. Stroke survivors may present with multiple fall risk factors, including hemiplegia, impaired balance, gait instability, sensory deficits, and fear of falling, highlighting the need for population‐specific evidence synthesis and implementation guidance. To address this gap, this evidence summary identified, appraised, and synthesized Chinese and international evidence on OEP‐based fall prevention after stroke, organizing the findings according to a clinical implementation pathway—comprising fall risk assessment, exercise intervention principles, OEP components, exercise prescription and implementation, safety management, health education, and intervention effects—to inform clinical nursing practice and community‐based continuing rehabilitation.

## 2 Methods

This study was conducted as a systematic, implementation‐oriented, evidence summary based on the 6S evidence model and the Joanna Briggs Institute (JBI) evidence appraisal approach. The protocol for this evidence summary was registered in PROSPERO (CRD420261418397). Ethical approval was not required for this evidence summary because it was based exclusively on previously published literature and did not involve human participants, personal data, or biological materials.

### 2.1 Formulation of the Evidence‐Based Question

The evidence‐based question was formulated using the Population, Intervention, Professional, Outcomes, Setting, and Type of evidence (PIPOST) framework (Zhu et al. 2017). The population included adults after stroke, including ischaemic and haemorrhagic strokes and post‐stroke hemiplegia. The intervention was the OEP or a modified OEP‐based programme, conducted alone or as a major component of a combined intervention. Outcomes included fall incidence, fall risk, fall‐related self‐efficacy, balance function, gait function, lower‐limb strength, mobility, activities of daily living, and quality of life. The intended users and settings included rehabilitation physicians and therapists, neurology nurses, and other healthcare professionals working in hospital, community, and home‐based continuing care contexts. Eligible evidence types included clinical practice guidelines, expert consensus documents, evidence summaries, clinical decision resources, practice standards, meta‐analyses, and randomized controlled trials (RCTs).

### 2.2 Search Strategy

The search was conducted according to the 6S evidence pyramid model (Alper and Haynes 2016), with guidelines searched first, followed by systematic reviews and primary studies. A systematic search was performed in relevant Chinese and international guideline repositories and databases, including the National Institute for Health and Care Excellence repository, the JBI Evidence‐Based Healthcare Library, UpToDate, the Cochrane Library, PubMed, Web of Science, Embase, CINAHL Ultimate, the Chinese Biomedical Literature Database, China National Knowledge Infrastructure (CNKI), Wanfang Data, and VIP Database. The reference lists of the included studies were manually searched as supplementary sources. Using PubMed as an example, the English search strategy was as follows:

#((“Stroke”[Mesh] OR “stroke”[tiab] OR “post‐stroke”[tiab] OR “cerebrovascular accident”[tiab])) AND ((“Accidental Falls”[Mesh] OR fall*[tiab] OR “fall prevention”[tiab] OR “fall risk”[tiab])) AND (“otago”[tiab] OR “otago exercise”[tiab] OR “otago exercise program”[tiab] OR “otago exercise programme”[tiab] OR “OEP”[tiab]). The literature search covered the period from the inception of each database to May 2026. CINAHL Ultimate was searched in October 2026, with the publication date restricted to May 2026 or earlier. The PubMed search strategy presented above primarily targeted OEP‐specific evidence in adults after stroke. Broader stroke rehabilitation and fall‐prevention guidance relevant to clinical implementation was identified through guideline repositories and supplementary reference searching.

### 2.3 Inclusion and Exclusion Criteria

The inclusion criteria were as follows: (1) OEP effectiveness studies included adults aged ≥ 18 years after stroke. Implementation‐related guidance could include broader populations. (2) Effectiveness studies involved OEP or modified OEP. During screening, eligibility was expanded to include relevant stroke rehabilitation and fall‐prevention guidance for implementation purposes, not as direct evidence of OEP effectiveness. (3) Outcomes included falls, balance, lower‐limb strength, fall‐related self‐efficacy, and relevant functional or implementation outcomes. (4) Eligible evidence included guidelines, expert consensus, evidence summaries, clinical decision resources, practice or group standards, systematic reviews/meta‐analyses, and RCTs. (5) Publications were in Chinese or English. During literature screening, the eligibility criteria were broadened to include relevant stroke rehabilitation and fall‐prevention guidance because of the limited availability of direct OEP evidence. This amendment was documented in the updated PROSPERO record.

The exclusion criteria were as follows: (1) studies in which participants had other neurological disorders potentially affecting balance function; (2) duplicate publications; (3) conference abstracts for which the full text was unavailable; and (4) studies with low methodological quality or insufficient evidence reliability.

### 2.4 Methodological Quality Appraisal

Methodological quality appraisal was conducted independently by two researchers who had received training in evidence‐based nursing. Clinical guidelines were appraised using the AGREE II instrument, which evaluates six domains—scope and purpose, stakeholder involvement, rigor of development, clarity of presentation, applicability, and editorial independence. Guidelines with standardized scores above 60% across all domains were rated as Grade A, while those with scores between 30% and 60% and scores below 30% were rated as Grades B and C, respectively. Only Grade A and Grade B guidelines were included in this study (Zhu et al. 2020). Expert consensus statements and meta‐analyses were appraised using the corresponding quality appraisal criteria developed by the JBI Evidence‐Based Healthcare Centre. Each item was rated “yes,” “no,” “unclear,” or “not applicable” (Hu and Hao 2018). RCTs were assessed using the 2023 JBI critical appraisal tool (Barker et al. 2023). For evidence summaries, original evidence sources were traced step by step and appropriate JBI appraisal tools were selected according to their study designs (Xing et al. 2020). If a traced primary study had already been identified through the present search, it was not included again but was used solely to verify the evidence source. If a traced primary study had not been identified through the present search but met the inclusion criteria, it was included as supplementary literature for methodological quality appraisal and evidence extraction. Group and practice standards were evaluated using the JBI checklist for textual evidence, which includes seven items, each of which was rated “yes,” “no,” “unclear,” or “not applicable” (Zhou et al. 2024). Any disagreement was resolved through discussion with a third researcher. When evidence sources overlapped or conclusions were inconsistent, the evidence was integrated by prioritizing higher‐quality evidence, evidence more directly relevant to the research question, and more recently published evidence.

### 2.5 Evidence Extraction and Grading

Two researchers independently reviewed the full texts and extracted relevant information. Extracted data included the author, year of publication, country, study design, study population, intervention, outcome indicators, and key findings. The level of evidence was graded according to the JBI evidence preclassification system, with Levels 1 and 5 representing the highest and lowest levels of evidence, respectively. Following discussions within the research team, the extracted evidence was organized according to the clinical implementation pathway for OEP‐based fall prevention in patients with stroke. The evidence was categorized into six major themes: fall risk assessment, principles of exercise intervention, core components of the OEP, exercise prescription parameters, safety screening and health education, and intervention effects of the OEP. A narrative synthesis was then conducted.

Where OEP‐specific evidence for adults after stroke was limited, evidence from closely related areas—including stroke rehabilitation, fall risk assessment, and fall‐prevention practice—was used to inform implementation‐focused evidence statements. In this context, high‐quality evidence was defined as evidence derived from methodologically rigorous studies with low risk of bias, as determined by the JBI critical appraisal tools or the AGREE II instrument. The directness of each evidence statement to OEP‐based fall prevention after stroke was considered during synthesis and interpretation. Direct OEP‐related evidence was used to inform intervention components and effects, whereas indirect stroke rehabilitation and fall‐prevention evidence was used only to inform implementation‐related recommendations. Indirect evidence was not interpreted as demonstrating OEP effectiveness.

### 2.6 Artificial Intelligence Generated Content Statement

During the preparation of this evidence summary, the authors used ChatGPT (OpenAI) between April 2026 and May 2026 for language refinement, expression polishing, and reference format standardization to improve text readability and fluency. The tool was applied to the following sections of the manuscript: the abstract, introduction, methods, results, discussion, and references. After the AI tool generated suggestions, all outputs were critically reviewed, fact‐checked, and edited by the authors to verify their accuracy, coherence, and compliance with the journal's style requirements. No sensitive, proprietary, or human subject data were uploaded to or processed by the AI tool. All core scientific work, including study design, literature screening, methodological quality appraisal, data extraction, evidence synthesis, statistical interpretation, and final conclusions, was independently completed by the author team. The AI tool was not used to generate any scientific data, research findings, or analytical decisions, nor was it listed as an author. The authors take full responsibility for the accuracy, integrity, and originality of the final manuscript.

## 3 Results

### 3.1 Literature Search Results

A total of 248 potentially relevant records were initially identified. After 140 duplicate records were removed using Zotero software, 108 records remained. Their titles and abstracts were screened, and records that did not satisfy the inclusion criteria for population, intervention, outcomes, or evidence type were excluded. Full texts of the remaining records were then assessed for eligibility. Studies that were inconsistent with the research topic, failed to meet quality appraisal criteria, or lacked available full texts were further excluded. Ultimately, 14 publications were included, comprising 3 clinical guidelines, 1 expert consensus statement, 2 practice standards, 1 evidence summary, 1 meta‐analysis, and 6 RCTs. The literature screening process is presented in Figure 1. The basic characteristics of the included studies are depicted in Table 1. Among the included RCTs, the study populations mainly comprised patients with stroke, including those with first‐ever cerebral infarction, cerebral infarction with hemiplegia, and sequelae of cerebral hemorrhage. These trials evaluated the OEP alone or the OEP combined with early rehabilitation. The methodological appraisal indicated that the included trials described the intervention protocols in terms of training frequency, session duration, intervention period, specific exercise components, and walking training requirements. The outcomes synthesized mainly involved balance function, mobility, fall‐related self‐efficacy, lower‐limb motor function or strength, activities of daily living, and quality of life. Reported functional measures included the Berg Balance Scale, Timed Up and Go (TUG) test, and the Modified Falls Efficacy Scale. Overall, the direct RCT evidence mainly supports the effects of OEP on fall‐related functional indicators, whereas evidence on actual fall incidence, number of falls, fall‐related injuries, and other hard clinical endpoints remains insufficient.

**FIGURE 1:**
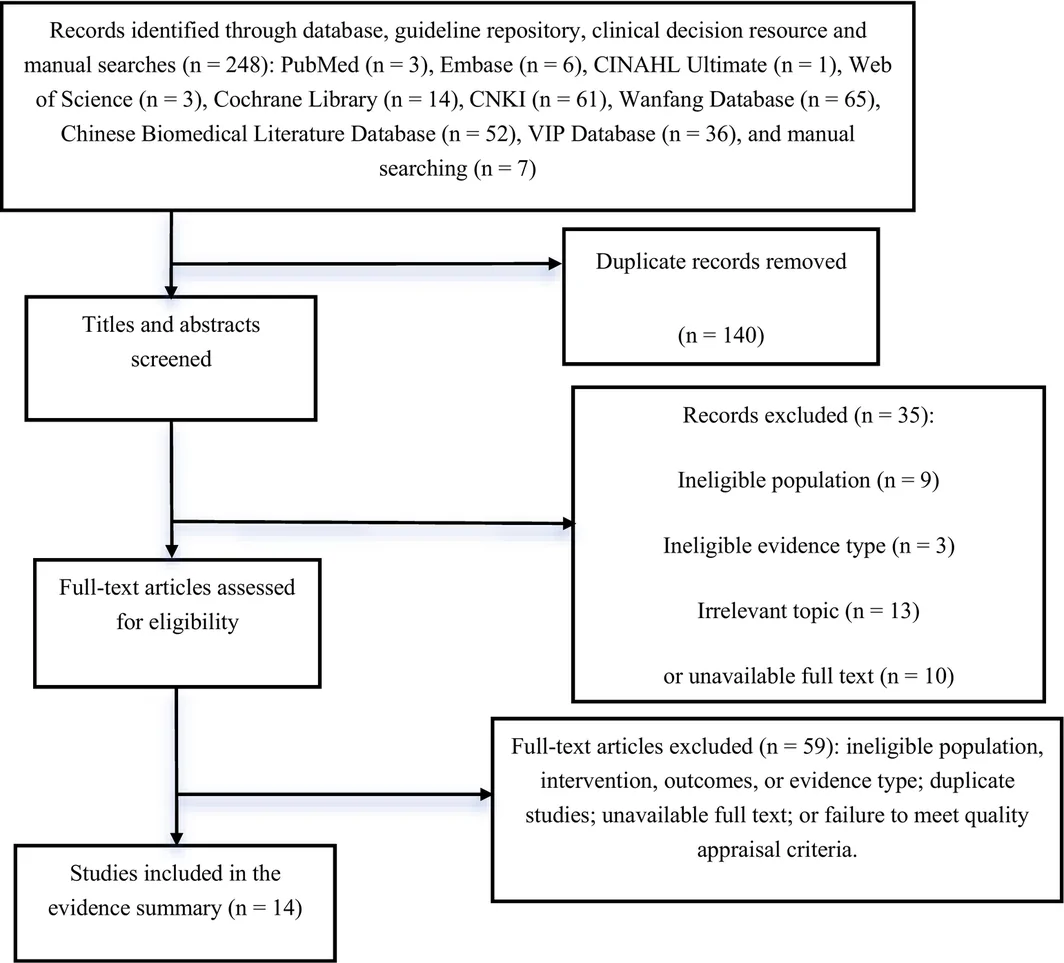
Flow diagram of the literature search and screening process. CNKI, China national knowledge infrastructure; Wanfang Data, Wanfang data; VIP, VIP information resource integration service platform.

**TABLE 1:** General characteristics of the included studies (*n* = 14).

Author(s)	Source of evidence	Type of evidence	Evidence topic	Year of publication
NICE	NICE	Guideline	Guideline for stroke rehabilitation in adults	2023
VA/DoD	VA/DoD	Guideline	Management of stroke rehabilitation	2024
Zhang et al.	CNKI	Guideline	Chinese guidelines for the clinical management of cerebrovascular diseases, second edition	2023
Expert consensus group on fall risk assessment and comprehensive intervention after stroke	CNKI	Expert consensus	Fall risk assessment and comprehensive intervention after stroke	2022
National health commission of the people's republic of China	Chinese national health commission	Practice standard	Technical standard for fall prevention and control among community‐dwelling older adults	2026
Chinese nursing association	Chinese nursing association	Group standard	Standard for fall risk assessment and prevention in adult inpatients	2020
Liu et al.	CNKI	Evidence summary	Exercise interventions for fall prevention in patients with stroke	2023
Pei et al.	CNKI	Meta‐analysis	Effects of the Otago Exercise Programme on fall‐related outcomes in patients with stroke	2019
Qiu	Wanfang Data	Randomized controlled trial	Preventive effect of the Otago Exercise Programme on fear of falling in patients with stroke	2016
Li et al.	CNKI	Randomized controlled trial	Effects of the Otago Exercise Programme on fall self‐efficacy and balance ability in patients with first‐ever cerebral infarction	2025
Xu	CNKI	Randomized controlled trial	Application effect of the OEP in acute rehabilitation among patients with first‐ever cerebral infarction	2020
M. M. Liu	CNKI	Randomized controlled trial	Application effect of Otago exercise training in older patients with hemiplegia after cerebral infarction	2022
Zhang	CNKI	Randomized controlled trial	Effects of the OEP combined with early rehabilitation on fall‐related function in patients with hemiplegia after cerebral infarction	2020
T. T. Liu et al.	CNKI	Randomized controlled trial	Effects of the OEP on motor dysfunction and fall‐related self‐efficacy in older patients with sequelae of cerebral hemorrhage	2022

Abbreviations: CNKI, China National Knowledge Infrastructure; NICE, National Institute for Health and Care Excellence; VA/DoD, U.S. Department of Veterans Affairs and the U.S. Department of Defense; Wanfang Data, Wanfang Data.

### 3.2 Quality Assessment Results of the Included Literature

#### 3.2.1 Guidelines

Three guidelines were included in this study (National Institute for Health and Care Excellence 2023; U.S. Department of Veterans Affairs and U.S. Department of Defense 2024; Zhang et al. 2023). Their domain‐specific appraisal results and recommendation grades are presented in Table 2.

**TABLE 2:** Quality assessment results of the included guidelines (*n* = 3).

Guideline	Standardized percentage score for each domain (%)						Number of domains with scores ≥ 60%	Number of domains with scores ≥ 30%	Recommendation level
	Scope and purpose	Stakeholder involvement	Rigor of development	Clarity of presentation	Applicability	Editorial independence			
NICE (2023)	90%	85%	95%	95%	75%	100%	6	6	Grade A
VA/DoD (2024)	92%	90%	93%	92%	85%	98%	6	6	Grade A
Zhang et al. (2023)	78%	55%	60%	82%	50%	65%	4	6	Grade B

Abbreviations: NICE, National Institute for Health and Care Excellence; VA/DoD, U.S. Department of Veterans Affairs and the U.S. Department of Defense.

#### 3.2.2 Expert Consensus

One expert consensus document was included (Expert Consensus Group on Fall Risk Assessment and Comprehensive Intervention After Stroke 2022). Following quality assessment, all appraisal items for this document were rated “yes”, indicating high overall quality. Its inclusion was therefore supported.

#### 3.2.3 Quality Assessment Results of the Practice and Group Standards

Two documents were included, comprising one practice and one group standard (Chinese Nursing Association 2020; National Health Commission of the People's Republic of China 2026). These were assessed using seven core appraisal items. The assessment results are presented in Table 3.

**TABLE 3:** Quality assessment results of the practice standard and group standard (*n* = 2).

References	①	②	③	④	⑤	⑥	⑦
National health commission of the people's republic of China (2026)	Yes	Yes	Yes	Unclear	Yes	Unclear	Yes
Chinese Nursing Association (2020)	Yes	Yes	Yes	Unclear	Unclear	Unclear	Yes

*Note:* The item numbers denote whether ① the source of textual evidence was clearly identified, ② the evidence source had professional authority in the relevant field, ③ the textual evidence considered the interests of the target population or relevant populations, ④ the conclusions were based on analytical results and supported by logical arguments, ⑤ relevant existing literature was referenced, ⑥ explanations or justifications were provided when the opinions were inconsistent with previous literature, and ⑦ the proposed opinions supported the final conclusions or recommendations.

#### 3.2.4 Evidence Summary

One evidence summary was included (Liu et al. 2023). Through stepwise tracing of its evidence sources, 11 source documents were identified, including 2 guidelines, 2 expert consensus documents, and 7 systematic reviews. Methodological quality assessment indicated that the guidelines were rated as Grade B overall. The expert consensus documents were of high overall quality. The systematic reviews were generally of moderate or high quality. The main methodological limitations included insufficient assessment of publication bias, incomplete reporting of search strategies, and substantial heterogeneity among the included studies in some systematic reviews. Overall, the source documents supporting this evidence summary were of moderate to above‐moderate quality. No clearly low‐quality or critically low‐quality literature was identified. Therefore, the synthesized evidence was considered to have reasonable credibility. Further tracing revealed that a systematic review and meta‐analysis directly evaluated the effects of the OEP on fall‐related outcomes in patients with stroke (Pei et al. 2019). This topic was highly consistent with the focus of the present study. Therefore, the review was included as systematic review‐level evidence related to the OEP, and the original RCTs within it were further traced.

#### 3.2.5 Meta‐Analysis

The methodological quality of the included meta‐analysis (Pei et al. 2019) was systematically assessed using 11 core appraisal items. The assessment results are presented in Table 4.

**TABLE 4:** Quality assessment results of the meta‐analysis (*n* = 1).

Reference	①	②	③	④	⑤	⑥	⑦	⑧	⑨	⑩	⑪
Pei et al. (2019)	Yes	Yes	Yes	Yes	Yes	Yes	Yes	Yes	No	Yes	Yes

*Note:* The item numbers denote whether ① the evidence‐based question was clearly defined, ② the eligibility criteria were appropriate, ③ the search strategy was rigorous and systematic, ④ the databases and evidence resources searched were adequate, ⑤ the quality assessment criteria or appraisal tools were appropriate for the type of literature, ⑥literature quality assessment was conducted independently by two or more reviewers, ⑦ measures were taken to minimize errors during data extraction, ⑧ the methods utilized to synthesize the study findings were appropriate, ⑨ potential publication bias was assessed, ⑩ practice recommendations were supported by the systematic review findings, and ⑪ reasonable directions or recommendations for future research were proposed.

#### 3.2.6 Randomized Controlled Trials (RCTs)

Six RCTs were included (Li et al. 2025; M. M. Liu 2022; T. T. Liu et al. 2022; Qiu 2016; Xu 2020; Zhang 2020). Reporting of participant retention and missing outcome data varied across the six RCTs. Li et al. (2025) reported five withdrawals among 60 enrolled participants, with 55 participants included in the final analysis. Qiu (2016) reported complete follow‐up, whereas information on participant retention and missing data handling was insufficiently detailed in several other studies. All studies provided detailed descriptions of the interventions delivered to the experimental and control groups. These descriptions included training frequency, session duration, intervention period, specific exercise components, and walking training requirements of the OEP. Baseline characteristics, such as age, disease duration, and affected side of hemiplegia, were generally balanced across groups, indicating between‐group comparability. No selective reporting of outcome indicators was detected, and the interventions were clearly described and reproducible. Regarding randomization methods, the studies by Xu (2020), M. M. Liu  (2022), Qiu (2016), Zhang (2020), and T. T. Liu et al. (2022) clearly used true random allocation. By contrast, Li et al. (2025) mentioned randomization but did not specify the method. Allocation concealment was rated “unclear” in all six studies. Blinding was difficult to implement because OEP is an active, participant‐engaged rehabilitation intervention. Therefore, all included studies were rated “no” for these items. In addition, none of the included studies clearly reported whether outcome assessors were blinded. Therefore, blinding of outcome assessors was rated “unclear” in all studies. Regarding sample size and safety reporting, none of the six RCTs clearly reported the basis for sample size estimation and none specified whether intervention‐related adverse events occurred during the study period. Overall, the methodological quality of the included RCTs was moderate. The assessment results are presented in Table 5.

**TABLE 5:** Quality assessment results of the randomized controlled trials (*n* = 6).

Included study	①	②	③	④	⑤	⑥	⑦	⑧	⑨	⑩	⑪	⑫	⑬
Qiu (2016)	Yes	Unclear	Yes	No	No	Unclear	Yes	Yes	Unclear	Yes	Yes	Yes	Yes
Li et al. (2025)	Unclear	Unclear	Yes	No	No	Unclear	Yes	Unclear	Unclear	Yes	Yes	Yes	Yes
Xu (2020)	Yes	Unclear	Yes	No	No	Unclear	Yes	Unclear	Unclear	Yes	Yes	Yes	Yes
M. M. Liu (2022)	Yes	Unclear	Yes	No	No	Unclear	Yes	Unclear	Unclear	Yes	Yes	Yes	Yes
Zhang (2020)	Yes	Unclear	Yes	No	No	Unclear	No	Yes	Unclear	Yes	Yes	Yes	Yes
T. T. Liu et al. (2022)	Yes	Unclear	Yes	No	No	Unclear	Unclear	Yes	Unclear	Yes	Yes	Yes	Yes

*Note:* The item numbers denote whether ① the random sequence generation method was appropriate, ② allocation concealment was strictly implemented, ③ baseline characteristics were comparable between groups, ④ participants were blinded, ⑤ intervention providers were blinded, ⑥ outcome assessors were blinded, ⑦ treatments and nursing care other than the core intervention were consistent across groups, ⑧ follow‐up data were complete with no missing data, ⑨ data analysis followed the intention‐to‐treat principle, ⑩ consistent outcome measurement methods were applied across groups, ⑪ the selected outcome measurement tools had satisfactory reliability and validity, ⑫ appropriate statistical methods were employed, and ⑬ the overall trial design was scientifically rigorous and appropriate.

### 3.3 Evidence Summary

Direct OEP‐specific evidence was primarily derived from the included meta‐analysis and RCTs, whereas several recommendations regarding fall risk assessment, safety management, health education, and continuity management were supported by guidelines, standards, or consensus documents related to stroke rehabilitation or fall prevention more broadly. Therefore, evidence statements were interpreted according to both the level of evidence and the directness of their relevance to OEP‐based fall prevention in patients with stroke. Based on the evidence content, evidence level, methodological quality, and clinical applicability of the included literature, the research team extracted, integrated, and summarized the evidence, developing 18 best‐evidence statements across the 6 domains of fall risk assessment, principles of exercise intervention, core exercise components of the OEP, exercise prescription and implementation, safety management and health education, and intervention effects of the OEP.

The evidence statements followed the sequence of clinical implementation: The first domain emphasized the need for multidimensional fall risk assessment before OEP implementation; the second and third clarified the exercise principles and core OEP components, including lower‐limb strengthening, balance training, and gait training; the fourth and fifth focused on individualized prescription, supervised progression, safety screening, health education, and continuity of care; and, finally, the sixth domain summarized intervention effects, indicating that current OEP‐related evidence mainly supports improvements in functional outcomes and fall‐related self‐efficacy, whereas evidence on actual fall incidence and fall‐related injuries remains limited. Details are presented in Table 6.

**TABLE 6:** Summary of best evidence for fall prevention using the Otago Exercise Programme in patients with stroke.

Theme	Evidence statement	Evidence level	Recommendation grade
I. Fall risk assessment	Fall risk in patients with stroke is multifactorial. Assessment should focus on decreased muscle strength, spasticity, sensory impairment, neglect, visual field defects, balance impairment, reduced attention, cognitive function, history of falls, medication use, and environmental factors (Chinese Nursing Association 2020; Expert Consensus Group on Fall Risk Assessment and Comprehensive Intervention After Stroke 2022; National Health Commission of the People's Republic of China 2026).	Level 5b	B
	Fall risk assessment in patients with stroke should not rely on a single assessment tool alone. Instead, comprehensive evaluation should integrate clinical judgment with two or more assessment tools, such as the Timed Up and Go (TUG) test, Berg Balance Scale (BBS), Swedish Postural Assessment Scale for patients with stroke (SwePASS), gait speed test, and the Falls Efficacy Scale/Modified Falls Efficacy Scale (FES/MFES) (Expert Consensus Group on Fall Risk Assessment and Comprehensive Intervention After Stroke 2022).	Level 5b	B
	TUG, BBS, SwePASS, gait speed testing, and FES/MFES may be used to assess balance ability, mobility, and fall‐related psychological status (Expert Consensus Group on Fall Risk Assessment and Comprehensive Intervention After Stroke 2022; Li et al. 2025; M. M. Liu 2022; T. T. Liu et al. 2022; Qiu 2016; Xu 2020; Zhang 2020).	Level 5b	B
	For inpatients, fall risk assessment should be performed or repeated at admission, transfer between departments, changes in clinical condition, initiation of high fall risk medications, after a fall, and before discharge for patients at high risk of falls (Chinese Nursing Association 2020).	Level 5b	A
	In community or home‐based rehabilitation settings, fall risk stratification may be conducted based on history of falls in the previous 12 months, perceived balance status, fear of falling, fall severity, frailty status, gait speed, and TUG performance (National Health Commission of the People's Republic of China 2026).	Level 5b	A
II. Principles of exercise intervention	For patients after stroke who are at risk of falls or have balance impairments, individualized exercise programmes incorporating balance training, lower‐limb strengthening, and gait training should be considered according to their functional status, rehabilitation goals, and safety needs (Liu et al. 2023; National Institute for Health and Care Excellence 2023; U.S. Department of Veterans Affairs and U.S. Department of Defense 2024; Zhang et al. 2023).	Level 5b	B
	Patients who regain walking ability after stroke should receive gait training to improve endurance and walking speed. When feasible, group circuit training and fall‐prevention education may also be incorporated (National Institute for Health and Care Excellence 2023).	Level 1a	B
	Exercise interventions should follow the principles of individualisation, progression, and safety. Training difficulty should be adjusted according to stroke type, severity of hemiplegia, muscle strength, balance function, gait stability, cardiopulmonary tolerance, and caregiving conditions (Expert Consensus Group on Fall Risk Assessment and Comprehensive Intervention After Stroke 2022; Li et al. 2025; M. M. Liu 2022; T. T. Liu et al. 2022; U.S. Department of Veterans Affairs and U.S. Department of Defense 2024; Xu 2020; Zhang 2020).	Level 5b	A
	Fall prevention should not rely solely on exercise training but should also incorporate comprehensive measures such as health education, medication management, use of assistive devices, environmental safety management, and post‐fall management (Chinese Nursing Association 2020; Expert Consensus Group on Fall Risk Assessment and Comprehensive Intervention After Stroke 2022; Liu et al. 2023; National Health Commission of the People's Republic of China 2026).	Level 5b	B
III. Core exercise components of the OEP	The OEP mainly includes lower‐limb strength training, balance training, and gait training, and may serve as an important component of exercise‐based fall prevention interventions for patients with stroke (Li et al. 2025; M. M. Liu 2022; T. T. Liu et al. 2022; Pei et al. 2019; Qiu 2016; Xu 2020; Zhang 2020).	Level 1c	B
	Lower‐limb strength training may include knee extension and flexion, hip abduction and extension, ankle dorsiflexion and plantar flexion, and sit‐to‐stand transitions, with an emphasis on improving lower‐limb support and postural control (Li et al. 2025; M. M. Liu 2022; T. T. Liu et al. 2022; Xu 2020; Zhang 2020).	Level 1c	B
	Balance training may include semi‐tandem standing, heel‐to‐toe standing, sideways walking, backward walking, turning exercises, single‐leg standing, and stepping exercises. Assistance, supervision, or reduced movement difficulty should be selected according to the patient's safety capacity (Li et al. 2025; M. M. Liu 2022; T. T. Liu et al. 2022; Qiu 2016; Xu 2020; Zhang 2020).	Level 1c	B
IV. Exercise prescription and implementation	The frequency, intensity, and duration of OEP training should be individualized according to the patient's functional status and should follow the principle of gradual progression (Li et al. 2025; M. M. Liu 2022; T. T. Liu et al. 2022; Pei et al. 2019; Qiu 2016; Xu 2020; Zhang 2020).	Level 5b	B
	During hospitalization, OEP may be implemented under the guidance of healthcare professionals. After discharge, the intervention may be continued through home‐based training, telephone or online follow‐up, and exercise logs to improve training adherence (Li et al. 2025; T. T. Liu et al. 2022; Xu 2020; Zhang 2020).	Level 1c	B
	For patients with marked balance impairment, a history of falls, or pronounced fear of falling, supervision and protection should be strengthened during the initial training stage. A gradual transition to independent home‐based training may be made after movement skills and safety capacity have improved (Chinese Nursing Association 2020; Expert Consensus Group on Fall Risk Assessment and Comprehensive Intervention After Stroke 2022; Li et al. 2025; M. M. Liu 2022; Qiu 2016).	Level 5b	A
V. Safety management and health education	Before implementing OEP, exercise safety screening should be conducted, paying particular attention to vital signs, level of consciousness, cardiopulmonary function, pain, fatigue, muscle tone, joint range of motion, gait and balance ability, and high‐risk factors for falls (Chinese Nursing Association 2020; Expert Consensus Group on Fall Risk Assessment and Comprehensive Intervention After Stroke 2022; Liu et al. 2023; National Health Commission of the People's Republic of China 2026).	Level 5b	A
	Individualized fall‐prevention education should be provided to patients and caregivers, covering fall risk identification, correct use of assistive devices, exercise precautions, ward or home environmental safety, medication precautions, recognition of abnormal symptoms, and post‐fall management (Chinese Nursing Association 2020; Expert Consensus Group on Fall Risk Assessment and Comprehensive Intervention After Stroke 2022; Liu et al. 2023; National Health Commission of the People's Republic of China 2026; National Institute for Health and Care Excellence 2023).	Level 5b	A
VI. Intervention effects of the OEP	OEP may improve fall‐related self‐efficacy, balance function, lower‐limb muscle strength, mobility, activities of daily living, and quality of life in patients with stroke. Combined interventions including OEP may also improve certain functional outcomes and fall‐related psychological status. Existing studies have mainly used functional outcomes and fall‐related self‐efficacy as primary outcomes, necessitating further evidence on actual fall incidence (Li et al. 2025; M. M. Liu 2022; T. T. Liu et al. 2022; Pei et al. 2019; Qiu 2016; Xu 2020; Zhang 2020).	Level 1a	B

*Note:* Evidence levels were determined according to the JBI 2014 Levels of Evidence, which indicate the hierarchy and type of evidence source rather than the certainty of the intervention effect. Level 1a denotes systematic reviews of multiple randomized controlled trials (RCTs); Level 1c, a single randomized controlled trial; Level 5b, expert consensus. Textual evidence, such as practice and group standards, was classified as Level 5b with reference to expert consensus or textual evidence. The methodological quality of guidelines was initially assessed using AGREE II. In the evidence summary table, when the original evidence supporting a guideline recommendation could be traced, the corresponding JBI evidence level was assigned. When the supporting evidence could not be sufficiently traced, the recommendation was conservatively classified as Level 5b, according to its nature as guideline‐based or expert opinion evidence. Grade A recommendations denote evidence that is highly relevant to fall prevention or OEP intervention in patients with stroke. Such evidence was derived from relatively high‐quality sources, showed generally consistent findings, involved patient safety management, or demonstrated good clinical feasibility. Grade B recommendations indicate that the evidence has supportive value but is limited by factors such as lower evidence levels, small sample sizes, indirectness of evidence, combined interventions, or inconsistency in outcome indicators.

## 4 Discussion

### 4.1 The OEP Has Positive Effects on Fall‐Related Functional Outcomes in Patients With Stroke

Although the broader evidence base for post‐stroke rehabilitation and fall prevention is relatively extensive, high‐level primary studies that directly evaluate OEP‐based interventions in patients with stroke remain scarce. The six included RCTs were of moderate overall quality. Their main limitations included unreported allocation concealment, difficulties in implementing blinding, and insufficient reporting of sample size estimation and intention‐to‐treat analysis (Li et al. 2025; M. M. Liu 2022; T. T. Liu et al. 2022; Qiu 2016; Xu 2020; Zhang 2020). Existing evidence suggests that the OEP may improve fall‐related functional outcomes in patients with stroke, including balance function, lower‐limb muscle strength, mobility, and fall‐related self‐efficacy. However, its effects on hard clinical outcomes—such as actual fall incidence, number of falls, and fall‐related injuries—require further validation in high‐quality studies.

### 4.2 The Training Components of the OEP Are Consistent With the Characteristics of Fall Risk in Patients With Stroke

The causes of falls in patients with stroke are complex. They often involve reduced lower‐limb muscle strength, impaired balance control, gait abnormalities, sensory deficits, cognitive or attentional decline, fear of falling, and environmental and medication‐related factors (Expert Consensus Group on Fall Risk Assessment and Comprehensive Intervention After Stroke 2022; Liu et al. 2023; National Health Commission of the People's Republic of China 2026). The core components of the OEP include lower‐limb strength training, as well as balance and gait training, which target several of the fall risk factors described above (Li et al. 2025; M. M. Liu 2022; T. T. Liu et al. 2022; Pei et al. 2019; Qiu 2016; Xu 2020; Zhang 2020). For example, knee extension and flexion exercises, as well as ankle dorsiflexion, may improve weight‐bearing capacity of the affected lower limb. Balance exercises—such as semi‐tandem or single‐leg standing and stepping exercises—may enhance static and dynamic balance. Gait training may improve mobility and gait stability during activities of daily living (Li et al. 2025; M. M. Liu 2022; T. T. Liu et al. 2022; Xu 2020; Zhang 2020).

The included RCTs reported improvements in one or more fall‐related outcomes—including balance, mobility and fall‐related self‐efficacy—although the specific outcome measures varied across studies (Li et al. 2025; M. M. Liu 2022; T. T. Liu et al. 2022; Qiu 2016; Xu 2020; Zhang 2020). Although these findings support the use of the OEP to target modifiable functional mechanisms of post‐stroke fall risk, they should not be interpreted as definitive evidence that the OEP reduces actual fall incidence or fall‐related injuries (Expert Consensus Group on Fall Risk Assessment and Comprehensive Intervention After Stroke 2022; Liu et al. 2023; National Health Commission of the People's Republic of China 2026).

### 4.3 Clinical Translation of the OEP Requires Individualized Prescription, Safety Screening, and Continuity of Care

The OEP is characterized by clear movements, graded training content, and suitability for supervised implementation during hospitalization and continued practice after discharge. However, patients with stroke often present with hemiplegia, abnormal muscle tone, sensory impairment, balance dysfunction, reduced cardiopulmonary tolerance, and fear of falling. Therefore, OEP frequency, intensity, movement difficulty, and intervention duration should be individualized according to stroke type, disease stage, severity of hemiplegia, muscle strength, balance function, gait stability, cardiopulmonary tolerance, and caregiving conditions (Expert Consensus Group on Fall Risk Assessment and Comprehensive Intervention After Stroke 2022). For patients in the acute or early recovery phase—or those with marked balance impairment, fear of falling, a history of falls or insufficient caregiving support—initial training should be supervised, with handrails, walking aids, or caregiver assistance provided when necessary (Li et al. 2025; T. T. Liu et al. 2022; Qiu 2016).

Before implementing the OEP, exercise safety screening should be performed, paying attention to vital signs, level of consciousness, cardiopulmonary function, pain, fatigue, muscle tone, joint range of motion, gait and balance ability, history of falls, high‐risk medications, and home environmental hazards (Chinese Nursing Association 2020; Expert Consensus Group on Fall Risk Assessment and Comprehensive Intervention After Stroke 2022; Liu et al. 2023; National Health Commission of the People's Republic of China 2026). During supervised training, nurses and rehabilitation professionals should monitor instruction comprehension, affected‐limb weight bearing, postural control, safe turning, and the appropriate use of handrails or walking aids. Dizziness, dyspnoea, chest discomfort, pain aggravation, marked fatigue, abnormal vital‐sign responses, loss of balance, or near‐falls should prompt reassessment of exercise difficulty and supervision needs.

For clinical translation, the OEP should be embedded within a structured nursing–rehabilitation pathway rather than provided as a simple list of exercises. During hospitalization, nurses may help identify modifiable fall risk factors, monitor exercise tolerance, and reinforce individualized fall‐prevention education. After discharge, home‐based OEP training should be supported by clear instructions, caregiver education, exercise logs, telephone or online follow‐up, and referral to community rehabilitation services when needed (Li et al. 2025; T. T. Liu et al. 2022; Qiu 2016; Zhang 2020). Therefore, the OEP should be implemented as an exercise‐based component of comprehensive fall‐prevention care and integrated with risk assessment, health education, environmental management, assistive‐device guidance, and continuity of care (Expert Consensus Group on Fall Risk Assessment and Comprehensive Intervention After Stroke 2022; Liu et al. 2023; National Institute for Health and Care Excellence 2023).

### 4.4 Strengths and Limitations

This study has several strengths. It focused specifically on the OEP as an exercise‐based fall‐prevention intervention for adults after stroke—a population with complex fall risk mechanisms and substantial rehabilitation needs. Evidence was searched and synthesized using the 6S evidence model, and multiple sources were included, including guidelines, consensus documents, practice standards, an evidence summary, a meta‐analysis, and RCTs. Evidence statements were organized according to a clinical implementation pathway, which may help nurses and rehabilitation teams translate available evidence into inpatient, community, and home‐based practice.

Several limitations should also be acknowledged. High‐quality primary studies directly evaluating the OEP in adults after stroke remain limited, and the included RCTs had methodological limitations, including insufficient reporting of allocation concealment, assessor blinding, sample size estimation, intention‐to‐treat analysis, and intervention‐related adverse events. During literature screening, the eligibility criteria were broadened to include indirect evidence from stroke rehabilitation and fall‐prevention guidance. Consequently, some evidence statements were supported by broader evidence rather than OEP‐specific studies, and several OEP‐based studies involved combined interventions. This may limit the direct applicability of some recommendations to OEP‐based fall prevention after stroke. In addition, most primary studies focused on functional outcomes, whereas evidence on fall incidence, recurrent falls, fall‐related injuries, and long‐term prognosis remains insufficient. These limitations should be considered when interpreting and implementing the evidence in clinical practice.

## 5 Conclusion

This study systematically identified, appraised, and synthesized evidence on the use of the OEP for fall prevention in adults after stroke. Eighteen best‐evidence statements were summarized across six domains: fall risk assessment, exercise intervention principles, core OEP components, exercise prescription and implementation, safety management and health education, and intervention effects.

Current evidence suggests that the OEP may improve balance function, lower‐limb muscle strength, mobility, fall‐related self‐efficacy, activities of daily living, and quality of life. These effects are clinically relevant because they reflect improvements in important functional and patient‐related outcomes after stroke. However, direct evidence confirming that the OEP independently reduces actual fall incidence or fall‐related injuries remains insufficient. Therefore, the OEP should be integrated as an exercise‐based component of comprehensive fall‐prevention care rather than as a stand‐alone fall‐prevention intervention.

Future studies should adopt rigorous, adequately powered, multicentre RCTs with longer follow‐up and fall‐related clinical outcomes, including fall incidence, recurrent falls, fall‐related injuries, and intervention‐related adverse events, to further evaluate the long‐term effectiveness and safety of the OEP in adults after stroke.

This evidence summary provides clinically applicable guidance for nurses involved in fall prevention and ongoing rehabilitation for adults after stroke. Fall risk assessment should be integrated into routine nursing care at admission, before discharge, and at key transition points. Nurses should use tools such as the TUG test and the Berg Balance Scale, together with clinical judgment, to identify patients at elevated fall risk and help formulate individualized fall‐prevention plans. The OEP may be implemented as a nurse‐facilitated exercise intervention during hospitalization in collaboration with rehabilitation physicians and therapists, with initial safety screening, exercise tolerance monitoring, and gradual progression tailored to each patient's functional status. Discharge planning should also include a structured continuity plan for home‐based OEP training, incorporating patient and caregiver education, written or digital exercise instructions, exercise logs, telephone or online follow‐up, and referral to community rehabilitation services to support adherence and safety after discharge. Nurses should also recognize that the OEP is a single component of a comprehensive fall‐prevention strategy rather than a stand‐alone solution. Therefore, exercise intervention should be integrated with environmental safety management, review of medication‐related fall risk, assistive‐device education, caregiver involvement, and patient education to support safer implementation across inpatient, community, and home settings.

## 6 Clinical Resources

- U.S. Department of Veterans Affairs and Department of Defense—Clinical Practice Guideline for the Management of Stroke Rehabilitation.

https://www.healthquality.va.gov/guidelines/Rehab/stroke/.

- American Heart Association/American Stroke Association—Guidelines for Adult Stroke Rehabilitation and Recovery.

https://www.ahajournals.org/doi/10.1161/STR.0000000000000098.

- Centers for Disease Control and Prevention—STEADI (Stopping Elderly Accidents, Deaths & Injuries): Fall Prevention Resources for Healthcare Providers.

https://www.cdc.gov/steadi/.

## Funding

The authors have nothing to report.

## Ethics Statement

Ethical approval was not required for this evidence summary because it was based exclusively on previously published literature and did not involve human participants, personal data, or biological materials.

## Consent

The authors have nothing to report.

## Conflicts of Interest

The authors declare no conflicts of interest.

## Data Availability

This study is an evidence summary based on published literature. All data supporting the findings of this study are available in publicly accessible databases.

## References

[jnu70157-bib-0001] Alper, B. S. , and R. B. Haynes . 2016. “EBHC Pyramid 5.0 for Accessing Preappraised Evidence and Guidance.” Evidence‐Based Medicine 21, no. 4: 123–125. 10.1136/ebmed-2016-110447. 27325531

[jnu70157-bib-0002] Barker, T. H. , J. C. Stone , K. Sears , et al. 2023. “The Revised JBI Critical Appraisal Tool for the Assessment of Risk of Bias for Randomized Controlled Trials.” JBI Evidence Synthesis 21, no. 3: 494–506. 10.11124/JBIES-22-00430. 36727247

[jnu70157-bib-0003] Chen, Y. , H. Du , M. Song , et al. 2023. “Relationship Between Fear of Falling and Fall Risk Among Older Patients With Stroke: A Structural Equation Modeling.” BMC Geriatrics 23, no. 1: 647. 10.1186/s12877-023-04298-y. PMC10568824 37821821

[jnu70157-bib-0004] Chinese Nursing Association . 2020. “Risk Assessment and Prevention of Falls in Adult Inpatients (Group Standard T/CNAS 18‐2020).”

[jnu70157-bib-0005] Expert Consensus Group on Fall Risk Assessment and Comprehensive Intervention After Stroke . 2022. “Expert Consensus on Fall Risk Assessment and Comprehensive Intervention After Stroke.” Journal of Clinical Internal Medicine 39, no. 1: 63–68.

[jnu70157-bib-0006] GBD 2021 Stroke Risk Factor Collaborators . 2024. “Global, Regional, and National Burden of Stroke and Its Risk Factors, 1990–2021: A Systematic Analysis for the Global Burden of Disease Study 2021.” Lancet Neurology 23, no. 10: 973–1003. 10.1016/S1474-4422(24)00369-7. PMC12254192 39304265

[jnu70157-bib-0007] Hu, Y. , and Y. F. Hao . 2018. Evidence‐Based Nursing. 2nd ed. People's Medical Publishing House.

[jnu70157-bib-0008] Li, Y. , Y. F. Song , W. N. Lu , et al. 2025. “Effects of the Otago Exercise Programme on Balance Ability and Fall Self‐Efficacy in Patients With First‐Ever Cerebral Infarction.” Chinese Journal of Geriatric Care 23, no. 6: 35–41. 10.3969/j.issn.1672-2671.2025.06.006.

[jnu70157-bib-0009] Liu, M. M. 2022. “Application Effect of Otago Exercise Training in Elderly Patients With Hemiplegia After Cerebral Infarction.” Medical Journal of Chinese People's Health 34, no. 18: 75–78. 10.3969/j.issn.1672-0369.2022.18.023.

[jnu70157-bib-0010] Liu, T. T. , Y. Gu , and X. D. Xu . 2022. “Effects of the Otago Exercise Programme on Motor Dysfunction in Elderly Patients With Sequelae of Cerebral Haemorrhage.” International Journal of Geriatrics 43, no. 5: 567–570. 10.3969/j.issn.1674-7593.2022.05.012.

[jnu70157-bib-0011] Liu, Y. M. , B. Q. Yan , and Y. H. Zhang . 2023. “Best Evidence Summary of Exercise Interventions for Fall Prevention in Stroke Patients.” Chinese General Practice Nursing 21, no. 10: 1297–1301. 10.12104/j.issn.1674-4748.2023.10.001.

[jnu70157-bib-0012] National Health Commission of the People's Republic of China . 2026. “Technical Standard for Fall Prevention and Control Among Community‐Dwelling Older Adults: WS/T 887–2026.” https://www.nhc.gov.cn/wjw/c100310/202603/1ec62b306fa9451a836e5a21fe1e2ce8.shtml.

[jnu70157-bib-0013] National Institute for Health and Care Excellence . 2023. Stroke Rehabilitation in Adults. NICE Guideline NG236. National Institute for Health and Care Excellence.

[jnu70157-bib-0014] Park, Y. , and M. Chang . 2016. “Effects of the Otago Exercise Program on Fall Efficacy, Activities of Daily Living and Quality of Life in Elderly Stroke Patients.” Journal of Physical Therapy Science 28, no. 1: 190–193. 10.1589/jpts.28.190. PMC4756001 26957755

[jnu70157-bib-0015] Pei, Z. W. , M. Wang , and X. M. Meng . 2019. “Effects of the Otago Exercise Programme on Fall Prevention in Stroke Patients: A Meta‐Analysis.” Rehabilitation Medicine 29, no. 2: 6–66. 10.3724/SP.J.1329.2019.02060.

[jnu70157-bib-0016] Qiu, H. Z. 2016. “Preventive Effect of the Otago Exercise Programme on Fear of Falling in Stroke Patients.” Journal of Chinese Physician 18, no. 5: 734–736. 10.3760/cma.j.issn.1008-1372.2016.05.027.

[jnu70157-bib-0017] U.S. Department of Veterans Affairs and U.S. Department of Defense . 2024. “VA/DoD Clinical Practice Guideline for the Management of Stroke Rehabilitation.”

[jnu70157-bib-0018] Winstein, C. J. , J. Stein , R. Arena , et al. 2016. “Guidelines for Adult Stroke Rehabilitation and Recovery: A Guideline for Healthcare Professionals From the American Heart Association/American Stroke Association.” Stroke 47, no. 6: e98–e169. 10.1161/STR.0000000000000098. 27145936

[jnu70157-bib-0019] Xing, W. J. , Y. Hu , Y. F. Zhou , et al. 2020. “Promoting the Translation of Evidence Into Clinical Practice (6): Development and Writing of Evidence Summaries.” Journal of Nurses Training 35, no. 12: 1129–1132. 10.16821/j.cnki.hsjx.2020.12.016.

[jnu70157-bib-0020] Xu, Q. Y. 2020. “Application of the Otago Exercise Programme in Acute Rehabilitation of Patients With First‐Ever Cerebral Infarction.” Master's thesis, Guangxi University of Chinese Medicine. 10.27879/d.cnki.ggxzy.2019.000255.

[jnu70157-bib-0021] Zhang, H. L. 2020. “Effects of the Otago Exercise Programme Combined With Early Rehabilitation on FMA Score, Lower‐Limb Muscle Strength and MFES Score in Patients With Hemiplegia After Cerebral Infarction.” Guangdong Medical Journal 41, no. 5: 491–495. 10.13820/j.cnki.gdyx.20193058.

[jnu70157-bib-0022] Zhang, L. , J. Wang , X. Dong , et al. 2025. “Injurious Falls Before, During, and After Stroke Diagnosis: A Population‐Based Study.” Journal of the American Medical Directors Association 26, no. 3: 105465. 10.1016/j.jamda.2024.105465. 39828242

[jnu70157-bib-0023] Zhang, T. , J. Zhao , X. P. Li , et al. 2023. “Chinese Stroke Association Guidelines for Clinical Management of Cerebrovascular Diseases (Second Edition) (Excerpt)—Chapter Eight: Cerebrovascular Disease Rehabilitation Management.” Chinese Journal of Stroke 18, no. 9: 1036–1048. 10.3969/j.issn.1673-5765.2023.09.010.

[jnu70157-bib-0024] Zhou, Y. F. , Y. Hu , Z. Zhu , et al. 2024. “JBI Critical Appraisal Checklist for Textual Evidence.” Journal of Nurses Training 39, no. 10: 1075–1080. 10.16821/j.cnki.hsjx.2024.10.012.

[jnu70157-bib-0025] Zhu, Z. , Y. Hu , W. J. Xing , Y. F. Zhou , and Y. Gu . 2017. “Composition of Different Types of Evidence‐Based Questions.” Journal of Nurses Training 32, no. 21: 1991–1994. 10.16821/j.cnki.hsjx.2017.21.025.

[jnu70157-bib-0026] Zhu, Z. , Y. Hu , Y. F. Zhou , et al. 2020. “Promoting the Translation of Evidence Into Clinical Practice (5): Methodological Quality Appraisal in Evidence Implementation Research.” Journal of Nurses Training 35, no. 11: 996–1000. 10.16821/j.cnki.hsjx.2020.11.009.

[jnu70157-bib-0027] Zhu, Z. , M. Shi , Q. Yu , et al. 2025. “Burden and Risk Factors of Stroke Worldwide and in China: An Analysis From the Global Burden of Disease Study 2021.” Chinese Medical Journal 138, no. 20: 2588–2595. 10.1097/CM9.0000000000003778. PMC12537097 40908884

